# The Influence of Cr_2_N Addition and Ni/Mn Ratio Variation on Mechanical and Corrosion Properties of HIP-Sintered 316L Stainless Steel

**DOI:** 10.3390/ma18122722

**Published:** 2025-06-10

**Authors:** Minsu Lee, Hohyeong Kim, Seok-Won Son, Jinho Ahn

**Affiliations:** 1 Industrial Components R&D Department, Korea Institute of Industrial Technology, Incheon 21999, Republic of Korea; lms0120@kitech.re.kr (M.L.); khh0524@kitech.re.kr (H.K.); 2 Department of Materials Science and Engineering, Hanyang University, Seoul 04763, Republic of Korea; 3 Department of Materials Science and Engineering, Inha University, Incheon 22212, Republic of Korea; 4 Customized Manufacturing R&D Department, Korea Institute of Industrial Technology, Incheon 21999, Republic of Korea

**Keywords:** chromium nitride, Ni/Mn ratio, hot isostatic pressing (HIP), 316L austenitic steel, nickel equivalent, microstructure

## Abstract

316L stainless steel is widely employed in various industrial sectors, including shipbuilding, offshore plants, high-temperature/high-pressure (HTHP) piping systems, and hydrogen infrastructure, due to its excellent mechanical stability, superior corrosion resistance, and robust resistance to hydrogen embrittlement. This study presents 316L stainless steel alloys fabricated via hot isostatic pressing (HIP), conducted at 1300 °C and 100 MPa for 2 h, incorporating Cr_2_N powder and an optimized Ni/Mn ratio based on the nickel equivalent (Ni_eq). During HIP, Cr_2_N decomposition yielded a uniformly refined, dense austenitic microstructure, with enhanced corrosion resistance and mechanical performance. Corrosion resistance was evaluated by potentiodynamic polarization in 3.5 wt.% NaCl after 1 h of OCP stabilization, using a scan range of −0.25 V to +1.5 V (Ag/AgCl) at 1 mV/s. Optimization of the Ni/Mn ratio effectively improved the pitting corrosion resistance and mechanical strength. It is cost-effective to partially substitute Ni with Mn. Of the various alloys, C13Ni-N exhibited significantly enhanced hardness (~30% increase from 158.3 to 206.2 HV) attributable to nitrogen-induced solid solution strengthening. E11Ni-HM exhibited the highest pitting corrosion resistance given the superior PREN value (31.36). In summary, the incorporation of Cr_2_N and adjustment of the Ni/Mn ratio effectively improved the performance of 316L stainless steel alloys. Notably, alloy E11Ni-HM demonstrated a low corrosion current density of 0.131 μA/cm^2^, indicating superior corrosion resistance. These findings offer valuable insights for developing cost-efficient, mechanically robust corrosion-resistant materials for hydrogen-related applications. Further research will evaluate alloy resistance to hydrogen embrittlement and investigate long-term material stability.

## 1. Introduction

Austenitic stainless steel (STS) exhibits excellent mechanical properties and corrosion resistance, and thus finds applications in a wide range of industries [1,2]. Among them, STS 316L is widely used in the fabrication of pipelines for shipbuilding, offshore plants, and high-temperature/high-pressure (HTHP) applications, owing to its outstanding mechanical stability, corrosion resistance, and hydrogen embrittlement (HE) resistance [3,4,5,6]. For typical HTHP pipelines, Cr-Mo alloy steels and Ni-based superalloys with superior creep resistance and high-temperature corrosion resistance are commonly employed. In hydrogen environments, austenitic stainless steels, duplex stainless steels, and Ni-based alloys are preferred due to their stable passive films and hydrogen compatibility [7,8,9,10]. However, since Ni, Cr, and Mo are expensive, cost-effective alternatives that maintain the desired mechanical and anti-corrosion properties need to be identified. Mn, which stabilizes the austenitic phase and enhances the mechanical strength and toughness under both ambient and cryogenic conditions, may serve as a cost-efficient alternative to Ni [11,12,13]. STS 316L is particularly suitable for saline, high-temperature, or hydrogen environments, as it exhibits strong corrosion resistance and mechanical strength due to the presence of Mo, low sensitization risk during welding owing to its low carbon content, low hydrogen diffusivity, which mitigates HE, and excellent resistance to stress corrosion cracking (SCC) due to its stable passive film [14,15]. The key alloying elements for enhancing the mechanical and corrosion properties of 316L STS include nickel (Ni), manganese (Mn), chromium (Cr), and nitrogen (N). Ni stabilizes the austenitic phase and imparts ductility and toughness while mitigating localized embrittlement under hydrogen environments. Mn contributes to solid solution strengthening and microstructural stability, thereby improving strength and ductility even at cryogenic temperatures, and also acts as a deoxidizer. Cr promotes the formation of a passive oxide film that elevates the corrosion potential and enhances resistance to SCC and crevice corrosion [16,17,18].

With the advancement of hydrogen-related industries, large-scale hydrogen storage and transport infrastructure have become essential. Materials used for hydrogen pipelines—especially under high-pressure conditions—must meet stringent performance requirements, particularly regarding HE resistance and hydrogen-induced cracking (HIC) [19,20,21,22,23,24]. Accordingly, precise alloy design and comprehensive evaluation of mechanical and electrochemical properties are essential for reliable performance under high-pressure hydrogen environments. In this context, optimization of the Ni equivalent (Ni_eq) plays a crucial role [25,26,27]. The Ni_eq, influenced by austenite-stabilizing elements such as Ni, Mn, C, and N, serves as a key indicator of austenite stability and HE resistance [28,29]. N, in particular, significantly increases the Ni_eq and stacking fault energy (SFE), stabilizing the austenitic matrix, enhancing dislocation glide, and suppressing twinning-induced deformation and hydrogen-assisted cracking [30,31].

Powder metallurgy (PM) has emerged as an advanced metal manufacturing route enabling precise control over alloy composition and complex geometries. Recently, powder-based additive manufacturing (AM) has gained traction due to its design flexibility, allowing the fabrication of intricate metal parts, particularly in high-value sectors such as biomedical and aerospace industries [32,33]. PM-based manufacturing techniques provide high adaptability for both simple and complex components, offering an integrated platform for simultaneous alloy design and process optimization [34]. Among these, hot isostatic pressing (HIP) enables the fabrication of dense, homogeneous alloys by applying high temperature and pressure, while minimizing oxidation and impurity formation [35,36,37]. HIP is thus well-suited for producing highly reliable metal components for extreme hydrogen environments.

This study proposes 316L-based STS alloys with tailored Ni_eq values, achieved by Cr_2_N powder addition and systematic variation in the Ni/Mn ratio, aiming to improve the mechanical and corrosion performance while reducing the material cost. Cr_2_N enabled quantitative nitrogen control, while the designed Ni/Mn ratio allowed fine-tuning of the phase stability. Although HIP is relatively expensive compared to conventional methods, its advantages in achieving dense, defect-free microstructures make it a suitable platform for evaluating the fundamental effects of alloy design. The proposed strategy thus offers a viable design pathway for developing cost-effective and high-performance stainless steels for hydrogen infrastructure systems.

## 2. Experimental Procedures

### 2.1. Alloy Design and HIP

This study presents 316L STS appropriate for hydrogen environments. Both the stability of the austenite phase and HE resistance were considered. The alloys were designed using the Ni_eq formula that incorporates the N level. The Ni_eq predicts the austenite phase stability; such stability is essential. The Ni_eq was calculated using the following formula [31]:

Ni_eq_ = Ni + 0.65 Cr + 0.98 Mo + 1.05 Mn + 0.35 Si + 12.6 C + 33.6 N (1)

Four alloys (C13Ni, C13Ni-N, E13Ni-LM, and E11Ni-HM) were prepared; their compositions are summarized in Table 1. C13Ni is a commercial 316L powder. The chemical composition of C13Ni was certified in the manufacturer’s certificate of analysis (CoA), which was based on inductively coupled plasma (ICP) spectroscopy, nitrogen/oxygen (NO) analysis, and carbon/sulfur (CS) analysis. C13Ni-N was fabricated by incorporating Cr2N powder into C13Ni. E13Ni-LM and E11Ni-HM were experimentally designed to modify the Ni/Mn ratio.

**Figure 1:**
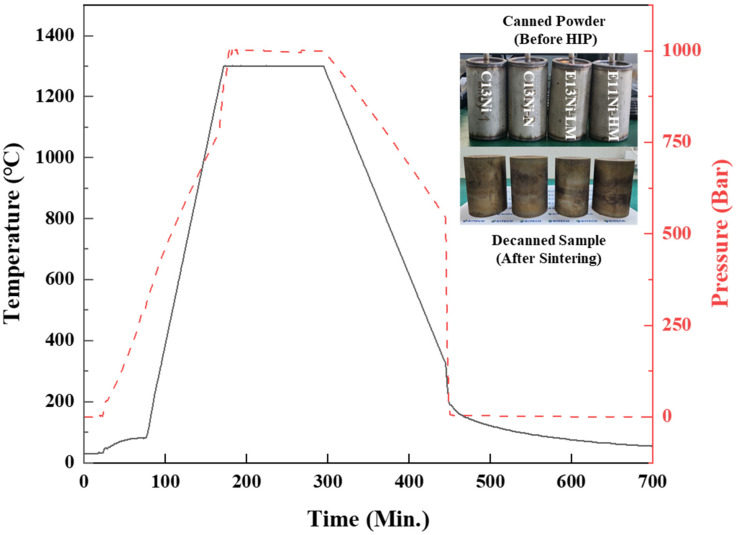
Hot isostatic press (HIP) temperature and pressure profiles, and images of canned powders and decanned sintered samples.

The Ni_eq of the commercial 316L alloy was 27.02. The addition of Cr_2_N powder and adjustment of the Ni and Mn contents yielded three alloys with Ni_eq values ≥ 29.63. C13Ni is a P-S316LS product of Konasol Co., Ltd. (Dangjin, Republic of Korea). This spherical alloy powder with D_max ≤ 149 μm is produced via gas atomization. C13Ni-N was prepared by adding Cr_2_N powder (Goodfellow, Cambs, UK) to C13Ni powder, followed by canning. The maximum particle size of Cr_2_N powder was 45 µm and the purity was 99.0%. Each elemental powder used in this study was of at least 99.0% purity. E13Ni-LM and E11Ni-HM were fabricated via gas atomization, which yielded spherical alloy powder with D_max ≤ 149 μm, followed by canning. After mixing, the powders were tapped into cans with dimensions of Φ76 × L120 mm, which were then degassed under a vacuum of approximately 10^−3^ torr, sealed, and the powders encapsulated. Sintering was performed using a hot isostatic press (HIP; AIP10-30H, American Isostatic Presses, Columbus, OH, USA) operating at 1300 °C and 1000 bar (100 MPa) for 2 h. The HIP heating and cooling rates were approximately 10 °C/min and 5 °C/min, respectively. After HIP, decanning proceeded via wire cutting and the alloys were fully characterized. The HIP temperature and pressure profiles are shown in Figure 1.

### 2.2. Characterization Methods

The fundamental properties of the four alloys were comprehensively evaluated. The chemical compositions were determined using glow discharge optical emission spectroscopy (GDOES; GDA 750 HR, Spectruma Analytik GmbH, Hof, Germany) to quantify the surface and internal Ni, Mn, Cr, Mo, C, Si, and N contents. The porosity characteristics of sintered alloy cross-sections were evaluated using the backscattered electron (BSE) imaging mode of a field-emission scanning electron microscope (SEM) (Apreo 2 S LoVac, Thermo Fisher Scientific, Waltham, MA, USA). The area fraction porosity was quantified with the aid of ImageJ 1.54g software (National Institutes of Health, Bethesda, MD, USA). Elemental composition employed an energy-dispersive spectrometer (EDS) (XFLASH 6160, Bruker, Mannheim, Germany). The microstructures were analyzed via electron backscatter diffraction (EBSD; Symmetry S2, Oxford Instruments, Abingdon, UK). All specimens were meticulously prepared using a focused ion beam (FIB; Nova NanoLab 600, FEI, Hillsboro, OR, USA) that enabled analysis of the grain structure and size distribution. X-ray diffraction (XRD; SmartLab, Rigaku, Tokyo, Japan) was employed to assess the stability of the austenite phase and identify phase transformations. Data were collected with the aid of Cu Kα radiation (λ = 1.5406 Å) over a 2θ range of 20–80°. Corrosion resistance was evaluated via potentiodynamic polarization tests adapted from ASTM G5-14 (2021) [38] and ASTM G61-86 (2018) [39] (PARSTAT 2273, Princeton Applied Research, Oak Ridge, TN, USA). The sintered specimens were surface-finished using 1200-grit SiC paper (Allied High Tech Products, Inc., Rancho Dominguez, CA, USA). Passivated specimens were held in a 20 wt.% nitric acid solution at room temperature for 1 h. Prior to testing, all specimens were stabilized for 1 h at their open-circuit potential (OCP). A 3.5 wt.% NaCl solution served as the electrolyte. All scans were conducted from −0.25 V to +1.5 V relative to a Ag/AgCl reference electrode. The scan rate was 1 mV/s. Following electrochemical analysis, pitted surfaces and sputtered areas revealed by GDOES were examined under a field-emission scanning electron microscope (FE-SEM; Apreo 2 S LoVac, Thermo Fisher Scientific). Bulk specimens after HIP were wire-cut into plate-shaped samples with initial thicknesses of 1.2, 1.5, 1.7, and 2.0 mm. The 1.2 mm thick specimens were used in the as-HIPed condition (0% reduction), while the others were cold-rolled to a final thickness of 1.2 mm, corresponding to total thickness reductions of 20%, 30%, and 40%, respectively. All rolling was conducted at room temperature using a four-high rolling mill without lubrication, and no intermediate annealing was applied. The thickness reduction per pass was controlled to be less than 15% to ensure uniform deformation. Mechanical properties were assessed using a Vickers hardness tester (HM-124, Mitutoyo, Kawasaki, Tokyo, Japan) and a load of 0.1 kgf (0.98 N). All measurements were repeated nine times, and the averages were calculated after excluding the highest and lowest values. These data were integrated to examine the combined effects of the grain size and the solid solution strengthening induced by compositional variation on the mechanical properties.

## 3. Results and Discussion

### 3.1. Chemical Composition and Microstructural Characterization

GDOES yielded the precise N contents of all four alloys. The calculated Ni_eq values and pitting resistance equivalent numbers (PRENs) are listed in Table 2. The PREN measures the contributions of Cr, Mo, and N to the corrosion resistance and is widely used to evaluate stability in hydrogen environments. The PRENs were calculated as follows [40,41]:

PREN = %Cr + 3.3 × %Mo + 16 × %N.(2)

Typically, Cr and Mo promote passive film formation and regeneration. N significantly increases the PREN, indicating enhanced corrosion resistance in chloride environments. The N interacts with the Cr, improving the passive film stability and mitigating hydrogen-induced degradation [42,43].

The designed and actual compositions (Table 1) exhibited consistent trends across elements, with only minor differences observed in certain cases. The increased carbon content compared to the design value was likely attributable to low-level carbon contamination during powder mixing or canning. As designed, only C13Ni-N contained added N. The Mn content increased from 0.07 wt% in C13Ni to 1.73 wt% in E13Ni-LM and 2.94 wt% in E11Ni-HM; the Mn content of C13Ni-N was similar to that of C13Ni at 0.08 wt%. E13Ni-LM and E11Ni-HM exhibited lower Ni and higher Mn contents than the other alloys, also as expected. The Ni_eq and PREN values of these two alloys were similar. The highest Ni_eq value (32.09) and PREN (31.36) were those of E11Ni-HM.

**Figure 2:**
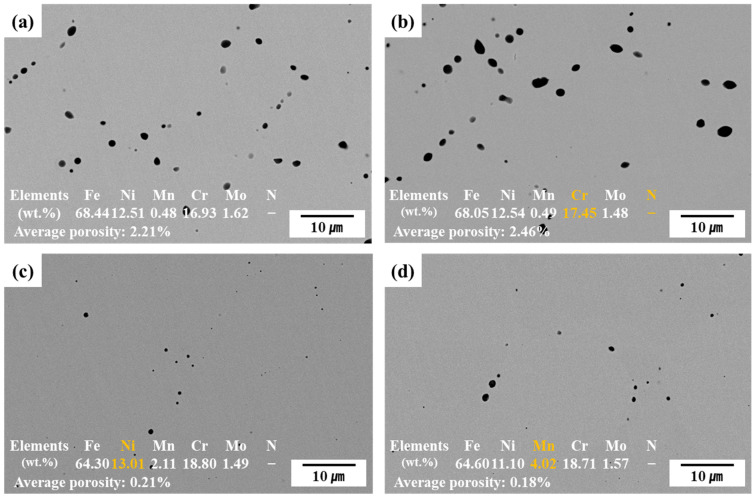
Cross-sectional scanning electron microscope (SEM) images of HIP-sintered 316L-based stainless steel (STS). (**a**) C13Ni, (**b**) C13Ni-N, (**c**) E13Ni-LM, (**d**) E11Ni-HM.

Figure 2 shows the SEM-BSE cross-sectional images and EDS-analyzed chemical compositions of the sintered alloys. The SEM images in Figure 2 were taken from the sectioned top surface of the HIP-sintered specimens. This surface corresponds to the one used for subsequent electrochemical corrosion testing. Black regions in the images represent pores formed during sintering. Compared to C13Ni and C13Ni-N alloys fabricated using commercial 316L powder, the newly designed alloys, E13Ni-LM and E11Ni-HM, exhibited significantly reduced porosity and more densified microstructures. The pores were primarily distributed along particle boundaries. Notably, many irregularly shaped pores were observed in C13Ni and C13Ni-N, whereas pores in E13Ni-LM and E11Ni-HM were relatively small and mostly spherical. The spherical morphology of these residual pores suggests that trace amounts of gas may have remained trapped during HIP consolidation, despite prior vacuum degassing at approximately 10^−3^ torr. In contrast, the presence of irregularly shaped pores may indicate non-gaseous origins such as insufficient particle bonding or shrinkage-induced cavities [44]. Although some micro-scale spherical pores were still observed in the HIP-processed samples, their size and frequency were significantly lower than those typically found in conventionally sintered steels. EDS analysis confirmed that the actual alloy compositions closely matched the designed compositions. However, N was not detected by EDS in alloy C13Ni-N.

**Figure 3:**
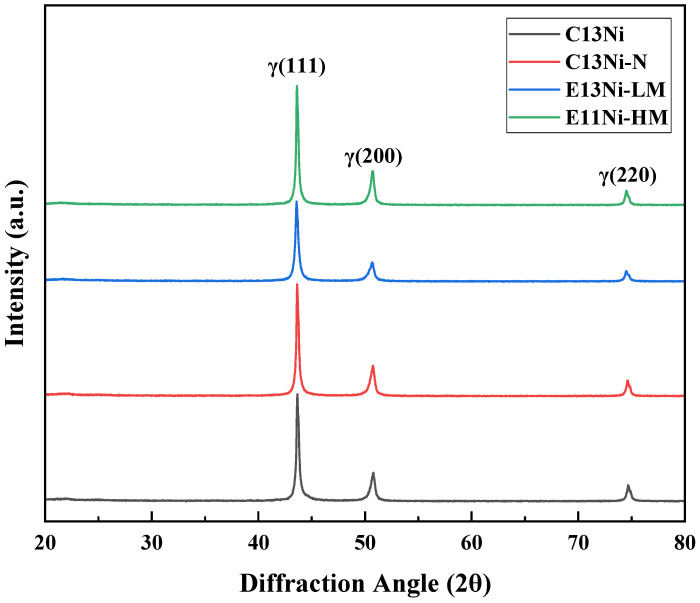
X-ray diffraction (XRD) patterns after HIP processing of 316L-based STS.

Figure 3 illustrates the XRD results. Phase analysis performed after HIP confirmed that the γ-austenite face-centered cubic (FCC) phase was the primary phase of all alloys. The Ni_eq values were approximately 25.85–31.36, ensuring the stability of the austenite phase and reinforcing the single-phase characteristics. The characteristic diffraction peaks were observed at (111)γ (2θ ≈ 43.5°), (200)γ (2θ ≈ 50.8°), and (220)γ (2θ ≈ 74.7°). Within the XRD detection limits, no secondary-phase peaks were observed, implying that austenite was the only phase present. This result and the generally high PRENs of the alloys imply that the corrosion resistance may have improved. The γ(111) peak positions of C13Ni-N and C13Ni were very similar. However, the γ(200) and γ(220) peak intensities varied upon the addition of Cr and N, both of which likely influenced the phase structure by exerting solid solution effects or inducing minor textural variations. For E13Ni-LM and E11Ni-HM, no significant differences in peak positions were observed, but the intensity ratio of the (111) and (200) peaks differed. E11Ni-HM exhibited a stronger γ(111) peak and a slightly weaker γ(200) peak, implying that textural development may have contributed to the intensity distribution differences. The overall similarity in the XRD patterns across different alloys indicates that the designed compositional modifications did not disrupt the single-phase γ-austenite structure. This confirms the microstructural stability of the alloys and provides a critical foundation for evaluating their mechanical and corrosion-related properties.

**Figure 4:**
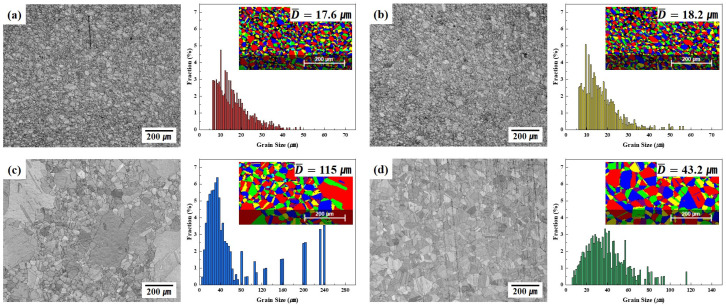
Electron backscatter diffraction (EBSD)-derived SEM images and grain size analysis of HIP-sintered 316L-based STS. (**a**) C13Ni, (**b**) C13Ni-N, (**c**) E13Ni-LM, (**d**) E11Ni-HM.

Figure 4 presents the SEM images and grain size distributions of the four alloys derived under optimal conditions for EBSD analysis. No significant macro-defects, such as large pores, extensive cracks, or heterogeneous regions, were observed in any sample. The images clearly demonstrate the effects of the various elements on both the grain boundary density and microstructural homogenization. The grains were generally equiaxed in shape; however, some samples exhibited relatively large grain size variation. Based on EBSD, the average grain sizes were 17.6 μm (C13Ni, Figure 4a), 18.2 μm (C13Ni-N, Figure 4b), 115 μm (E13Ni-LM, Figure 4c), and 43.2 μm (E11Ni-HM, Figure 4d). C13Ni and C13Ni-N, which had lower Cr and Mo contents than the other alloys, exhibited more uniform and smaller grains. Cr and Mo may have contributed to grain boundary stabilization through the possible formation of minor carbide or nitride precipitates, which likely acted as pinning sites that suppressed grain growth. In contrast, the substantially greater grain size of E13Ni-LM was attributable to the high Ni and lower Mn content; the Ni content was particularly elevated, at 13.07 wt%, and some grains were larger than 100 μm (Figure 4c, lower panel). The relatively low Mn content of E13Ni-LM likely limited the formation of fine precipitates, rendering pinning insufficient to suppress grain boundary movement. Consequently, a coarser grain structure was observed. Additionally, interaction between Ni and Mn may have enhanced grain boundary mobility. Although the Mn content of E11Ni-HM was higher than that of E13Ni-LM, the significantly greater Cr and Mo contents likely contributed to grain growth suppression, resulting in a smaller grain size. This finding implies that interactions among Ni, Mn, Cr, and Mo collectively influenced the grain size (Figure 4d, lower panel). The E11Ni-HM alloy exhibited more suppressed grain growth than E13Ni-LM, but still formed larger grains than those of the C13Ni series. The high Cr and Mo contents likely hindered grain boundary migration and reduced the growth rate, whereas Mn promoted diffusion and influenced the final grain size. Thus, the balance between Mn and stabilizing elements such as Cr and Mo is critical in terms of grain size control and microstructural uniformity. HIP enhances the density and uniformity, but achieving the abovementioned balance is key if 316L-based STS alloys are to be mechanically strong and corrosion-resistant.

**Figure 5:**
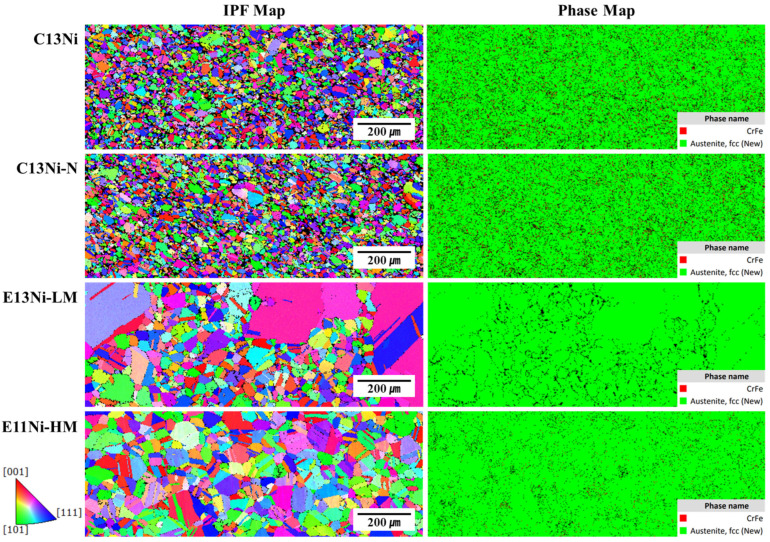
EBSD phase analyses and inverse pole figure (IPF) maps of HIP-sintered 316L-based STS.

Figure 5 shows the Z-direction EBSD inverse pole figures (IPFs) and phase maps of the sintered samples. All alloys principally exhibited the austenitic FCC phase; porosity was minimal, and the grain orientations favored the (111), (101), and (001) planes. Black areas in the phase maps were primarily attributable to pores or non-indexed regions associated with local surface defects, rather than unidentified phases. Such random textures imply that the mechanical properties may be isotropic. The principal microstructures were equiaxed austenite grains, often with annealing twins. Large and small grains were apparent. In stainless steels, Cr- and Fe-rich intermetallic compounds such as the sigma (σ) phase may form during prolonged exposure to elevated temperatures. These phases can degrade the ductility and toughness, while depleting Cr from the matrix and thus reducing the corrosion resistance, particularly against pitting and intergranular corrosion [45,46]. The phase maps revealed notable fractions of red-highlighted CrFe phases, particularly in C13Ni (3.86%) and C13Ni-N (4.15%), where CrFe was detected at a slightly higher level. However, the minor CrFe signals observed in the EBSD maps were likely attributable to local Cr enrichment within the FCC matrix rather than the formation of a separate secondary phase. This interpretation is consistent with the XRD results (Figure 3), where no distinct secondary-phase peaks were detected, confirming the single-phase austenitic structure of the alloys. Conversely, CrFe formation in E13Ni-LM was nearly absent, implying minimal phase segregation. In contrast, the CrFe level in E11Ni-HM (~0.84%) likely reflects localized Cr enrichment within the FCC matrix rather than a separate phase. Mo suppressed the formation of brittle phases, including the sigma (σ) phase, in line with the XRD results. C13Ni exhibited small, uniformly distributed, highly equiaxed grains, indicating effective recrystallization during sintering. C13Ni-N exhibited a fine, uniform grain structure similar to that of C13Ni, likely attributable to the stabilizing, solid solution strengthening effects of N. E13Ni-LM and E11Ni-HM exhibited larger grains, as shown in the IPF maps. The distinct color differences indicate variation in grain orientation and size. E13Ni-LM contained a mixture of coarse and fine grains, with the former confined to certain regions. The grain size of E11Ni-HM was more uniform than that of E13Ni-LM, but somewhat more heterogeneous than that of C13Ni. The predominance of intermediately sized grains in E11Ni-HM implies that recrystallization and grain growth occurred simultaneously. Despite the high Mn content of E11Ni-HM, the Cr and Mo content maintained the structural uniformity of that alloy. HIP controlled grain growth well and promoted uniform recrystallization. Despite differences in the Ni, Mn, Cr, and N contents, all samples were typical STS alloys that retained stable austenitic (FCC) structures post-sintering. A stable microstructure ensures corrosion resistance and ductility. Among all samples, C13Ni and C13Ni-N exhibited the most uniform grain structures. E13Ni-LM exhibited localized coarse grain growth and structural heterogeneity, implying vulnerability in mechanical performance. However, the stability of the austenitic phase and the minimal Cr-enriched phases imply that the material properties remained unaffected by such minor anomalies.

### 3.2. Corrosion Resistance and Mechanical Properties

**Figure 6:**
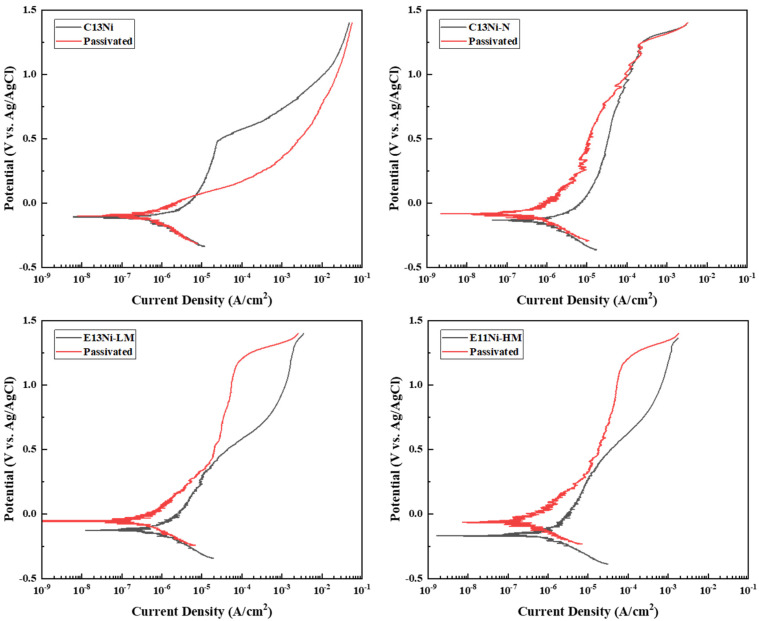
Effects of alloy compositions on the electrochemical corrosion potential of 316L-based STS in as-prepared and passivated states.

Figure 6 shows the potentiodynamic polarization curves of the alloys. The electrolyte was 3.5 wt.% NaCl. The cathodic polarization curves primarily reflect the reduction of dissolved oxygen in the NaCl electrolyte, which is typical for austenitic stainless steels exposed to chloride-containing environments. Within the measured potential range, the gradual increase in the cathodic current density suggests a possible contribution from the hydrogen evolution reaction (HER) in addition to oxygen reduction [47,48]. Although hydrogen-related degradation was not the main focus of this study, such cathodic behavior may be indirectly relevant to hydrogen embrittlement (HE), as pitting corrosion can create localized acidic environments that facilitate hydrogen ingress. In contrast, a stable passive film can suppress pit formation and thereby reduce the likelihood of hydrogen absorption, potentially mitigating HE susceptibility. The corrosion resistance of the four alloys was evaluated, and the additional effect of nitric acid passivation on passive film formation was analyzed by comparing sintered and passivated samples. Although oxide layers can form naturally during sintering, they often lack chemical uniformity and structural coherence. Therefore, post-passivation was conducted to promote the development of a chemically homogeneous and structurally stable passive film, providing a consistent basis for evaluating localized corrosion resistance in chloride environments.

**Figure 7:**
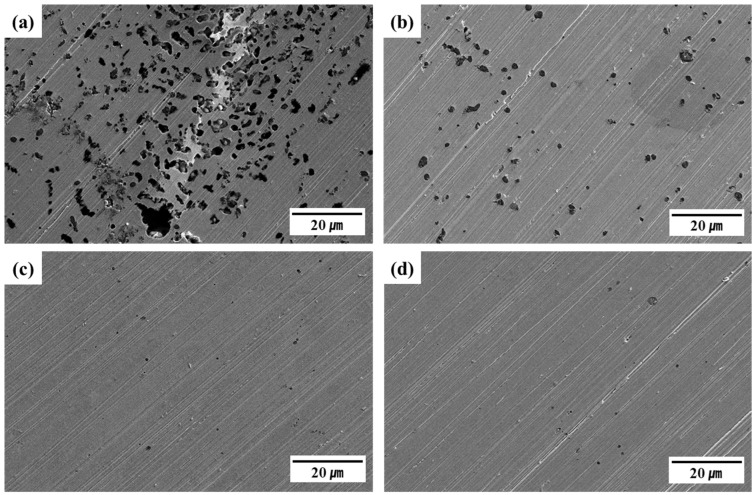
SEM-based pitting corrosion evaluation of 316L-based STS after potentiostatic polarization. (**a**) C13Ni, (**b**) C13Ni-N, (**c**) E13Ni-LM, (**d**) E11Ni-HM.

The corrosion potential (E_corr_), corrosion current density (I_corr_), and pitting potential (E_pit_) were determined using Tafel extrapolation. In terms of corrosion potentials measured by Tafel analysis, C13Ni exhibited the highest corrosion potential (−109 mV), indicating relatively noble behavior. However, the corrosion current density (0.519 μA/cm^2^) was intermediate among those of the tested alloys. Notably, alloy E11Ni-HM exhibited the lowest corrosion potential (−172 mV) and the lowest corrosion current density (0.131 μA/cm^2^). This alloy demonstrated superior overall corrosion resistance despite its more negative corrosion potential. Alloys C13Ni-N and E13Ni-LM exhibited similar corrosion potential (−134 and −128 mV, respectively) but the highest corrosion current densities (0.867 and 0.872 μA/cm^2^, respectively). Consequently, these alloys were more susceptible to corrosion than were C13Ni and E11Ni-HM. Nitric acid passivation shifted the corrosion potentials of all alloys toward more noble values. However, the effects on the corrosion current density varied among alloys. In particular, alloys C13Ni-N and E13Ni-LM exhibited significantly decreased corrosion current densities after passivation (from 0.867 to 0.510 μA/cm^2^ for C13Ni-N and from 0.872 to 0.348 μA/cm^2^ for E13Ni-LM). Passivation clearly improved the corrosion resistance. Conversely, alloys C13Ni and E11Ni-HM exhibited increased corrosion current densities after passivation (from 0.519 to 0.631 μA/cm^2^ for C13Ni and from 0.131 to 0.326 μA/cm^2^ for E11Ni-HM), implying that although passivation shifted the potentials positively, it may have partially disrupted or weakened pre-existing passive films, in association with somewhat higher corrosion kinetics. Additionally, the substantial increase in pitting potentials for alloys such as E13Ni-LM and E11Ni-HM highlights improved resistance to localized corrosion. These results demonstrate that passivation alters the surface oxide characteristics and can either enhance or deteriorate the corrosion performance depending on the alloy composition. The polarization curves indicate pseudopassivation behavior, characterized by the absence of a well-defined passive current plateau and relatively high current densities within the assumed passive region. Alloys such as C13Ni-N and E13Ni-LM exhibited poor passive film stability, as indicated by their high corrosion current densities (0.867 and 0.872 μA/cm^2^, respectively). Specifically, E13Ni-LM, with its large grain size (115 μm), may have had reduced grain boundary density, limiting passive film coverage. In contrast, E11Ni-HM exhibited the lowest current density (0.131 μA/cm^2^), attributed to the formation of a robust Cr_2_O_3_-based film enabled by its high Cr (21.82 wt.%) and Mo (2.89 wt.%) contents. A sharp increase in current beyond the pitting potential (E_pit_) indicates the onset of transpassivation and passive film breakdown. Such behavior observed in passivated C13Ni, particularly the absence of a passive region and elevated I_corr_, is indicative of a pseudopassivation mechanism rather than the formation of a stable passive film. Nitric acid treatment likely disrupted the native oxide layer, resulting in a thin or porous passive film that was prone to early breakdown. Moreover, the low PREN value (25.85), attributed to the absence of nitrogen and only moderate Cr (18.29 wt%) and Mo (2.29 wt%) contents, may have further limited the stability of the passive layer in chloride environments. In contrast to C13Ni-N, where 0.09 wt% nitrogen enhanced the passive film stability through Cr–N interactions, the absence of nitrogen in C13Ni appears to have exacerbated film degradation. Furthermore, although the CrFe phase (3.86%) detected by EBSD may have induced local Cr depletion and weakened the passive film integrity, its role is likely secondary to nitrogen deficiency, as C13Ni-N (4.30% CrFe) exhibited improved passivation behavior. These combined effects are reflected in the substantial reduction in the E_pit_ from 479 mV to 48 mV, confirming the compromised passivation stability of C13Ni. To clarify the reliability of the E_pit_ values, it should be noted that for E13Ni-LM and E11Ni-HM, the anodic current increased gradually without a sharp inflection point. In these cases, the E_pit_ was conservatively estimated at the onset of sustained current rise. At high potentials above 1.2 V (vs. Ag/AgCl), additional reactions such as transpassivation and oxygen evolution may contribute to current increase. To address this, the post-polarization SEM images (Figure 7) were examined to verify actual pitting. The minimal surface damage observed in E11Ni-HM supports the validity of the estimated E_pit_.

Although C13Ni exhibited the lowest PREN (25.85) among the four alloys, its pitting potential (479 mV) was higher than those of E13Ni-LM (407 mV) and E11Ni-HM (424 mV), as shown in Table 3. This inverse trend suggests that pitting resistance is influenced not only by the PREN but also by microstructural features such as the grain boundary density and passive film integrity. Additionally, due to the indistinct transition and gradual current rise in the polarization curves (Figure 6), graphical determination of the E_pit_ for E13Ni-LM and E11Ni-HM may involve uncertainty. Thus, caution is advised when comparing E_pit_ values solely based on polarization plots. While this inverse trend is evident in C13Ni, a general correlation between a higher PREN and increased pitting potential was still observed in the other alloys, such as E11Ni-HM. E13Ni-LM and E11Ni-HM were expected to exhibit similar corrosion resistance given their comparable Ni_eq and PREN values. However, E13Ni-LM exhibited relatively poor overall corrosion resistance, attributable to significantly enlarged grains, which reduced the total grain boundary area and thus sites favoring rapid formation and stabilization of the protective passive film. Consequently, the passive film that formed on E13Ni-LM was less stable, increasing susceptibility to corrosion. The higher N content and PREN (27.74) of C13Ni-N enhanced the passive film stability. However, the relatively high corrosion current density indicated that the addition of a small amount of N was not as effective as expected in terms of improving the overall corrosion resistance. The different current densities in the passive regions further highlight the variations in corrosion resistance among the alloys.

Figure 7 shows the surface morphologies of specimens after the potentiodynamic polarization tests, as revealed by SEM. All specimens exhibited multiple pits near the grain boundaries, reflecting active dissolution. Clear differences in the pit density, size, and distribution were observed among the alloys, closely related to their electrochemical corrosion characteristics. Although the corrosion current densities (I_corr_, 0.131–0.872 μA/cm^2^) of the four alloys in the polarization results (Figure 6) were relatively similar, pitting corrosion is a localized phenomenon that is more sensitively influenced by the PREN value, alloy composition, and microstructural features. These factors contributed to the pronounced differences in surface morphology observed in Figure 7. The C13Ni alloy exhibited extensive pitting corrosion, with many large pits uniformly distributed across the entire surface. C13Ni-N exhibited less corrosion than C13Ni, attributable to the effect of the Cr_2_N addition to C13Ni, which reduced the pit density and size. In contrast, E13Ni-LM and E11Ni-HM exhibited minimal surface corrosion. These alloys demonstrated significantly enhanced resistance to localized corrosion, with markedly fewer, smaller pits. Among the tested alloys, E11Ni-HM exhibited the smallest pits and lowest pit densities, reflecting the best corrosion performance, consistent with its superior electrochemical properties. This excellent resistance in E11Ni-HM can be attributed to its high PREN (31.36) and elevated contents of Cr (21.82 wt%) and Mo (2.89 wt%), which promoted the formation of a stable passive film and effectively suppressed pitting (Figure 7d). In C13Ni-N, the addition of nitrogen (0.09 wt%) enhanced the passive film stability and reduced pitting compared to C13Ni (Figure 7b). Although E13Ni-LM possessed a relatively large grain size (115 μm), which reduces the grain boundary area and potential pit initiation sites, its high PREN (30.76) contributed to maintaining good pitting corrosion resistance (Figure 7c). The high corrosion resistance of 316L-based STS alloys was primarily attributable to the presence of Ni and Mo. Alloys with higher PRENs exhibited better resistance to localized corrosion. Electrochemical tests revealed that variation in the Cr, Mn, Ni, and N contents significantly affected the corrosion resistance. The SEM observations clearly supported the polarization test results, confirming that the alloy composition and microstructural characteristics significantly influence the corrosion behavior. The observed pit morphologies are consistent with transpassive degradation beyond the pitting potential. C13Ni exhibited extensive and widespread pitting, suggesting early transpassive breakdown of the passive film. In contrast, E11Ni-HM showed minimal surface damage, highlighting its superior film stability even under high anodic polarization.

**Figure 8:**
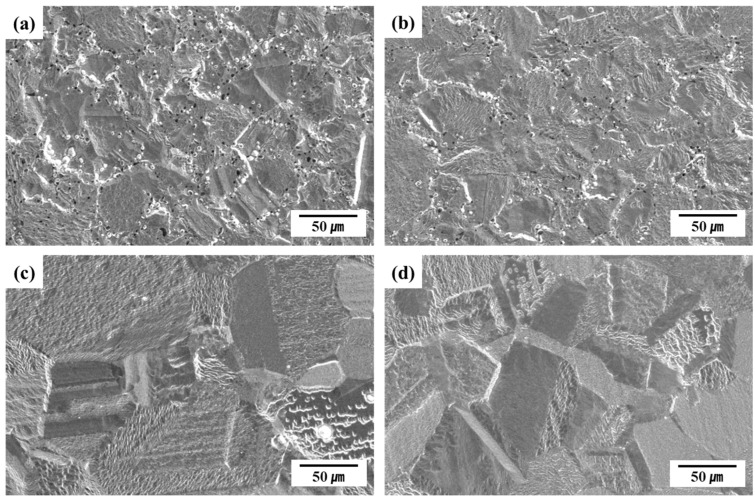
SEM-revealed surface morphologies of 316L-based STS subjected to glow discharge optical emission spectroscopy (GDOES) analysis. (**a**) C13Ni, (**b**) C13Ni-N, (**c**) E13Ni-LM, (**d**) E11Ni-HM.

Figure 8 presents the SEM surface images obtained after quantitative analysis using GDOES. Plasma etching was employed to clearly reveal the microstructural features and surface morphology. The latter, and pitting, were subsequently evaluated via SEM, and the results were compared to those of the potentiodynamic polarization tests. E13Ni-LM and E11Ni-HM exhibited minimal pitting, whereas C13Ni and C13Ni-N demonstrated clear pitting near the grain boundaries. In particular, C13Ni exhibited extensive localized etching along these boundaries, clearly indicating susceptibility to localized corrosion. C13Ni-N exhibited reduced but notable etching near the grain boundaries, reflecting the limited improvement afforded by N addition. Thus, based on the extent of the observed etching, C13Ni was identified as the alloy most susceptible to corrosion. The SEM images clearly revealed larger grain structures in E13Ni-LM and E11Ni-HM, which were well correlated with their superior surface uniformity and minimal etching damage. Plasma etching removed all surface oxide layers from E13Ni-LM and E11Ni-HM, resulting in highly uniform surfaces. Such morphological uniformity is indicative of stable surface properties. Additionally, E11Ni-HM demonstrated particularly clear, stable grain boundary morphologies, reinforcing their excellent corrosion resistance and uniform surface properties. The surface morphology after plasma etching was in line with the results obtained after the potentiodynamic polarization tests.

**Figure 9:**
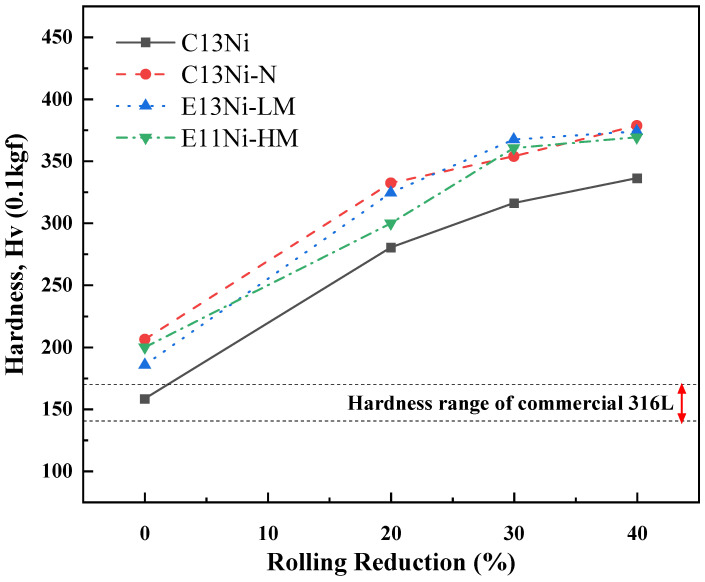
Vickers hardness values of alloys designed using the Ni_eq as a function of rolling reduction.

Figure 9 presents the results of the Vickers hardness tests to evaluate the mechanical properties of the alloys, which were compared with the literature-reported hardness of commercial 316L austenitic stainless steel sheets [49,50]. The hardness values varied depending on the Ni_eq and the compositional differences. The hardness value of C13Ni was comparable to that of the commercial cold-rolled 316L sheet, as reported in the previous literature. C13Ni, with the smallest average grain size (17.6 μm), had the lowest value (158.3 HV); this was because the solid solution strengthening effect was poor due to the low contents of strengthening elements such as Ni, Mn, Cr, and N. As the rolling reductions increased from 0 to 40%, all alloys exhibited significantly increased hardness due to the work-hardening effects induced by plastic deformation. However, the increase in hardness observed in C13Ni was comparatively limited. Even at a 40% rolling reduction, its hardness (approximately 320 HV) remained the lowest among all alloys tested.

In contrast, E13Ni-LM and E11Ni-HM had larger grain sizes, at 115 and 43.2 μm, respectively. The increase in the Mn content from 1.73 wt% to 2.94 wt% contributed to solid solution strengthening. The hardness value of E13Ni-LM was 185.4 HV; that of E11Ni-HM was 199.7 HV. The higher Mn content and smaller grain size of E11Ni-HM enhanced the hardness via solid solution strengthening. At high rolling reductions (30–40%), the hardness values of E13Ni-LM and E11Ni-HM converged, indicating similar work-hardening behaviors despite their initial grain size differences. C13Ni-N, which had a grain size of 18.2 μm, similar to that of C13Ni, exhibited the highest average hardness value of 206.2 HV, attributable to solid solution strengthening by N and microstructural stabilization by both Cr and N. Notably, C13Ni-N exhibited the most pronounced increase in hardness at lower rolling reductions (20%), demonstrating that N addition significantly enhanced the work-hardening capability. Thus, the Ni, Mn, Cr, and N proportions influenced both the hardness and microstructural stability. N crucially enhanced the hardness of C13Ni-N via solid solution strengthening. The combined effects of Mn, Cr, and N on the microstructural stability emphasize that careful design is essential to optimize the mechanical properties of 316L STS.

The composition and interaction of alloying elements (Cr, Mo, Ni, Mn, and N) played a decisive role in enhancing both the corrosion resistance and hardness of 316L stainless steels, as quantitatively verified by the EBSD analyses (Figure 4 and Figure 5) and the electrochemical and mechanical test results (Figure 6, Figure 7, Figure 8 and Figure 9). Cr and Mo promoted the formation of stable Cr_2_O_3_-based passive films, effectively suppressing the pitting corrosion in chloride environments, while N synergistically stabilized the passive film and increased the PREN (e.g., 31.36 for E11Ni-HM, approximately 5.5 higher than C13Ni). Despite its moderate grain size (43.2 μm, Figure 4d), E11Ni-HM exhibited superior corrosion resistance with only minor pitting (Figure 7d), owing to its high Cr (21.82 wt%) and Mo (2.89 wt%) contents. In contrast, E13Ni-LM had coarse grains (115 μm, Figure 4c), which reduced the grain boundary density and delayed passive film regeneration, resulting in aggravated pitting (Figure 7c). The phase maps (Figure 5) confirmed the stable FCC austenite structure in all alloys, with E11Ni-HM showing a minimal CrFe phase fraction (0.84%), indicating effective suppression of harmful secondary phases. In terms of the hardness, the microstructural features and alloy compositions were closely correlated. Nitrogen contributed to solid solution strengthening and microstructural stabilization of the austenite matrix, resulting in the highest hardness (206.2 HV) for C13Ni-N with a fine grain size of 18.2 μm (Figure 4b), which represented a ~30% increase compared to C13Ni (158.3 HV). E11Ni-HM also maintained a high hardness of 199.7 HV despite its coarser grains, due to Mn-induced solid solution strengthening and Cr–Mo grain boundary pinning effects. Conversely, E13Ni-LM exhibited the lowest hardness (185.4 HV) among the high-Mn and high-Cr alloys due to insufficient grain boundary strengthening. These findings suggest that not only Hall–Petch grain refinement, but also solid solution strengthening, austenite phase stability, and suppression of deleterious phases collectively govern the corrosion and mechanical performance of 316L-based stainless steels. This study demonstrated, through a quantitative linkage between EBSD-based microstructural analysis and electrochemical/mechanical behaviors, that microstructure-controlled alloy design using HIP sintering is an effective strategy to develop durable 316L stainless steels suitable for extreme environments such as high-pressure hydrogen applications.

## 4. Conclusions

This study presents 316L stainless steel alloys fabricated via HIP by incorporating nitrogen through Cr_2_N powder addition, with systematic adjustment of the Ni/Mn ratio based on the nickel equivalent (Ni_eq). During HIP, the Cr_2_N powder decomposed, forming a refined austenitic microstructure that improved both the mechanical performance and corrosion resistance. The optimized Ni/Mn ratios in E13Ni-LM and E11Ni-HM resulted in higher PRENs and enhanced structural stability. These alloys exhibited better mechanical and electrochemical properties compared to the commercial C13Ni alloy. C13Ni-N, with 0.09 wt% N, exhibited the highest hardness (206.2 HV), approximately 30% greater than that of C13Ni (158.3 HV), due to solid solution strengthening and microstructural stabilization. E11Ni-HM demonstrated superior corrosion resistance, recording the lowest corrosion current density (0.131 μA/cm^2^) and the highest pitting potential (1308 mV), enabled by high Cr (21.82 wt%) and Mo (2.89 wt%) contents. Its PREN value (31.36) was 21% higher than that of C13Ni (25.85), highlighting the critical role of Cr and Mo in passive film stabilization. The results also confirm that partial substitution of Ni with Mn (up to 2.94 wt%) effectively maintained both the mechanical strength and corrosion resistance. Microstructural analysis showed that compositional adjustments significantly influenced the grain growth and phase stability. In summary, the combination of Cr_2_N addition and Ni/Mn ratio optimization successfully enhanced the performance of 316L-based alloys in a cost-effective manner. These findings provide design guidance for the development of durable, hydrogen-compatible stainless steels. Future work will evaluate their long-term resistance to hydrogen embrittlement under high-pressure hydrogen environments.

## Figures and Tables

**Table 1:** Compositions of 316L-based alloys designed using the nickel equivalent (Ni_eq).

Sample	Elements (wt.%)							Ni_eq
	Ni	C	Si	Mn	Cr	Mo	N	
C13Ni	13.05	0.02	0.90	0.04	17.10	2.35	-	27.02
C13Ni-N	13.05	0.02	0.90	0.04	18.30	2.35	0.10	31.23
E13Ni-LM	13.30	0.02	0.50	1.70	17.70	2.70	-	29.63
E11Ni-HM	11.30	0.02	0.50	3.70	17.70	2.70	-	29.76

**Table 2:** Glow discharge optical emission spectroscopy (GDOES)-measured compositions, calculated Ni_eq values, and pitting resistance equivalent numbers (PRENs).

Sample	Elements (wt.%)							Ni_eq	PREN
	Ni	C	Si	Mn	Cr	Mo	N		
C13Ni	13.29	0.06	0.99	0.07	18.29	2.29	-	28.60	25.85
C13Ni-N	13.01	0.07	0.99	0.08	19.30	2.12	0.09	31.97	27.74
E13Ni-LM	13.07	0.04	0.41	1.73	20.93	2.98	-	32.06	30.76
E11Ni-HM	11.34	0.04	0.40	2.94	21.82	2.89	-	32.09	31.36

**Table 3:** Potentiodynamic polarization parameters of the as-prepared and passivated alloys in 3.5 wt.% NaCl solution.

	E_corr_ (mV vs. Ag/AgCl)		I_corr_ (μA/cm^2^)		E_pit_ (mV vs. Ag/AgCl)	
	As-Prepared	Passivated	As-Prepared	Passivated	As-Prepared	Passivated
C13Ni	−109	−100	0.519	0.631	479	48
C13Ni-N	−134	−83	0.867	0.510	1227	1219
E13Ni-LM	−128	−53	0.872	0.348	407	1170
E11Ni-HM	−172	−63	0.131	0.326	424	1160

## Data Availability

The original contributions presented in this study are included in the article. Further inquiries can be directed to the corresponding authors.
